# Leg muscle assessment by HR-pQCT and DXA: associations between imaging measures and muscle function in adults with obesity—a pilot study

**DOI:** 10.1093/jbmrpl/ziag158

**Published:** 2026-09-22

**Authors:** Søren Gam, Joshua R Stapleton, Vivian Jing, Eric Zeng, S Delanie Lynch, Nina Z Heilmann, Kristin Cattell, Lori S Cogdill, Catherine MacLean, Sherri A Ford, Marjorie J Howard, Daniel P Beavers, Jamy D Ard, Steven K Boyd, Ashley A Weaver, Kristen M Beavers

**Affiliations:** Department of Endocrinology, Esbjerg and Grindsted Hospital, University Hospital of Southern Denmark, 6700 Esbjerg, Denmark; Department of Sports Science and Clinical Biomechanics, University of Southern Denmark, 5230 Odense M, Denmark; Department of Regional Health Research, University of Southern Denmark, 5230 Odense M, Denmark; Department of Biomedical Engineering, Wake Forest University School of Medicine, Winston-Salem, NC 27101, United States; Department of Health and Exercise Science, Wake Forest University, Winston-Salem, NC 27109, United States; Department of Biomedical Engineering, Wake Forest University School of Medicine, Winston-Salem, NC 27101, United States; Department of Biomedical Engineering, Wake Forest University School of Medicine, Winston-Salem, NC 27101, United States; Department of Biomedical Engineering, Wake Forest University School of Medicine, Winston-Salem, NC 27101, United States; Department of Biomedical Engineering, Wake Forest University School of Medicine, Winston-Salem, NC 27101, United States; Department of Health and Exercise Science, Wake Forest University, Winston-Salem, NC 27109, United States; Department of Health and Exercise Science, Wake Forest University, Winston-Salem, NC 27109, United States; Department of Health and Exercise Science, Wake Forest University, Winston-Salem, NC 27109, United States; Department of Biostatistics and Data Science, Wake Forest University School of Medicine, Winston-Salem, NC 27157, United States; Department of Statistical Sciences, Wake Forest University, Winston-Salem, NC 27109, United States; Weight Management Center, Atrium Health Wake Forest Baptist, Winston-Salem, NC 27104, United States; Department of Epidemiology and Prevention, Wake Forest University School of Medicine, Winston-Salem, NC 27157, United States; McCaig Institute for Bone and Joint Health, Cumming School of Medicine, University of Calgary, Calgary, AB T2N 4Z6, Canada; Department of Biomedical Engineering, Schulich School of Engineering, University of Calgary, Calgary, AB T2N 4Z6, Canada; Department of Radiology, Cumming School of Medicine, University of Calgary, Calgary, AB T2N 4Z6, Canada; Department of Biomedical Engineering, Wake Forest University School of Medicine, Winston-Salem, NC 27101, United States; Department of Health and Exercise Science, Wake Forest University, Winston-Salem, NC 27109, United States; Department of Internal Medicine, Section of Gerontology and Geriatric Medicine, Wake Forest University School of Medicine, Winston-Salem, NC 27157, United States

**Keywords:** obesity, DXA, HR-pQCT, muscle function, skeletal muscle, muscle mass

## Abstract

DXA and HR-pQCT are used for bone assessment but also image adjacent soft tissue. This pilot study examined association between HR-pQCT-measured leg muscle cross-sectional area (CSA) and density vs DXA-derived lean mass, and explored associations with muscle strength, power, and physical performance in adults with obesity. Patients eligible for bariatric surgery (BMI ≥ 30 kg/m^2^; ages ≥30) received baseline HR-pQCT (XtremeCT II) and DXA (Hologic) scans. HR-pQCT scans of the non-dominant leg were acquired at 30% of tibial length and processed using a custom Python script for muscle segmentation, followed by manual editing. Whole-body DXA scans were analyzed to extract lean mass for the total leg and subregions analogous to the HR-pQCT. Functional measures included gait speed (400-m walk), muscle power (12-step stair climb), and knee extensor strength (isokinetic dynamometer). Pearson correlations assessed relationships between imaging modalities and functional measures. Among 28 adults (ages: 47.7 ± 8.6; BMI: 43.8 ± 4.6 kg/m^2^), HR-pQCT muscle CSA and density averaged 36.0 ± 4.8 cm^2^ and 60.1 ± 8.1 HU, respectively. DXA lean mass was 2.23 ± 0.40 kg for the total leg and 0.107 ± 0.019 kg for the 30% site. HR-pQCT muscle CSA correlated moderately with DXA lean mass (*r* = 0.57-0.69, *p* = .0017-<.0001), whereas HR-pQCT muscle density showed negligible correlations with DXA lean mass (*r* = −0.01-0.04, *p* = .84-0.96). Significant positive correlations were observed for DXA total leg lean mass with absolute muscle power (*r* = 0.40) and HR-pQCT muscle CSA with knee strength (*r* = 0.41; both *p* < .05). In adults with obesity, HR-pQCT and DXA may provide complementary measures of leg muscle quantity, with moderate association for CSA and lean mass, but limited associations with muscle density. Body weight and whole-body lean mass showed stronger correlations with stair climb power (*r* = 0.42-0.70) than localized leg muscle measures (*r* = 0.17-0.40). Muscle density and muscle quantity may capture different aspects of musculoskeletal health, warranting further investigation in larger samples.

## Introduction

Obesity is associated with impaired relative muscle strength, which contributes to reduced physical function, mobility limitations, increased fall risk, and poorer quality of life.^1^ Consequently, many individuals pursue weight loss (diet, glucagon-like peptide-1 (GLP-1) receptor agonists, or bariatric surgery) to improve physical function and reduce the burden of obesity-related diseases. However, rapid weight reduction achieved through bariatric surgery or the increasingly accessible GLP-1 receptor agonists is often accompanied by 11%-20% losses of lean body mass (LBM), representing 20%-40% of total weight loss,^2–6^ and 16%-42% declines in absolute lower limb strength.^5^^,^^7–10^ Therefore, monitoring changes in muscle is warranted during surgical and pharmacological weight loss treatments to evaluate intervention effects and understand their long-term musculoskeletal consequence and effects on physical function and quality of life.

Several imaging modalities are available to assess skeletal muscle in both clinical and research settings. MRI and CT are generally considered as the gold standards, providing highly accurate and direct measurements of muscle quantity and quality.^11^^,^^12^ Conventional CT is typically used to assess muscle at central skeletal sites (eg, trunk and thigh) and provides high spatial resolution along with attenuation-based measures of muscle density. However, both modalities have several limitations, such as high cost, time consumption, and, in the case of CT, exposure to significant ionizing radiation.^11^ Therefore, DXA is often used for assessing lean mass, and it is widely applied in studies involving individuals with obesity.^2–6^^,^^13^^,^^14^^,^^15^ DXA is rapid, relatively inexpensive, and involves minimal radiation exposure (<5 μSv) compared to CT. DXA does not directly measure muscle mass but estimates LBM from whole-body scans by emitting X-rays at 2 different energy levels to distinguish bone and fat; subtraction of these components yields the lean mass estimate.^11^ Moreover, DXA has demonstrated strong reliability for assessing LBM and fat mass in adults with obesity, with coefficients of variation (CV) of 1.7% and 1.6%, respectively.^16^^,^^17^ In addition to whole-body composition, DXA also allows for regional analyses (eg, leg, mid-thigh, or whole leg), providing localized estimates of LBM. In adults with obesity, whole-body or appendicular DXA-derived LBM may be influenced by excess adiposity and include a substantial proportion of non-contractile tissue, potentially obscuring relationships between muscle morphology and functional outcomes. In older adults, DXA-derived LBM at these sites has been shown to correlate strongly with CT-measured muscle quantity.^18^ Regional muscle assessments may therefore provide additional insight beyond whole-body measures by enabling evaluation of muscle quality, improving anatomical matching with functional outcomes, and detecting localized muscle changes associated with aging, obesity, or weight loss interventions. However, regional DXA assessments are rarely performed^18^^,^^19^ and have not been well examined in adults with obesity or directly compared with other imaging modalities, which warrants further investigation.

HR-pQCT is an imaging technique that enables precise assessment of bone microarchitecture in the tibia and radius.^20^ While HR-pQCT was originally developed for bone analyses, the scan region also includes the surrounding soft tissue. This enables quantification of leg muscle cross-sectional area (CSA) and density, which have been shown to correlate with pQCT measurements in adults without obesity (CSA *R*^2^ = 0.95; density *R*^2^ = 0.66).^21^ A few studies have applied HR-pQCT to examine bone microarchitectural deterioration and bone loss following weight loss,^22–26^ but muscle mass has not yet been assessed with HR-pQCT in a population with obesity. HR-pQCT acquires 3D images at high spatial resolution (60.7 μm voxel size) with relatively low radiation exposure (<5 μSv) compared to conventional CT. However, there may be technical challenges when scanning individuals with obesity. Increased soft tissue thickness is known to affect HR-pQCT bone measurements by increasing measurement error and reducing precision, as greater soft tissue thickness increases X-ray attenuation and beam hardening, thereby degrading signal quality and potentially affecting image segmentation.^27^ Soft-tissue-related measurement artifacts have also been reported for DXA. The limited field of view of the scanner (140 mm) can also be challenging in individuals with very large legs. All of these issues are likely to similarly affect muscle measurements.

Although MRI is considered the gold standard for assessing skeletal muscle quantity and composition, DXA remains the most widely used clinical method for evaluating body composition and LBM due to its accessibility, low radiation exposure, and relatively low cost. DXA is a well-established method for assessing LBM, and DXA-derived lean mass parameters (eg, total lean mass, appendicular lean mass [ALM], and ALM/height^2^) show moderate associations with muscle strength in adults.^28^ Therefore, DXA provides an important reference against which HR-pQCT muscle parameters can be compared. HR-pQCT, in contrast, enables site-specific assessment of muscle morphology at peripheral skeletal sites while simultaneously capturing bone microarchitecture. Recently, a study by Warden et al. demonstrated that HR-pQCT-derived muscle density shows modest but statistically significant positive associations with grip strength, maximal gait speed, and chair-rise performance.^29^ Taken together, examining associations between HR-pQCT-derived muscle parameters and commonly used DXA-derived lean mass measures may help to clarify how site-specific HR-pQCT muscle measurements relate to DXA-derived lean mass at regional and whole-body levels, and whether these measures are relevant for understanding muscle function and physical performance in adults with obesity.

Therefore, the purpose of this pilot study was to investigate the relationship between leg muscle measures obtained using DXA and HR-pQCT in adults with obesity. Specifically, HR-pQCT muscle parameters were compared with DXA-derived lean mass at the 30% relative offset distance (computed by percent of the limb length from the distal tibia plafond of the non-dominant leg). Second, the study examined the associations of HR-pQCT muscle and DXA lean mass metrics with measures of muscle function and physical performance. We hypothesized that HR-pQCT muscle mass and density would be positively correlated with DXA lean mass, and that all of these measures would be positively correlated with muscle function and physical performance.

## Materials and methods

### Study design and participants

This cross-sectional pilot study used baseline data from participants with obesity (BMI ≥ 30 kg/m^2^) aged 30 yr or older who were bariatric surgery candidates enrolled in an ongoing, randomized, double-blind, placebo-controlled clinical trial (NCT04922333). The trial protocol has previously been published.^30^ A convenience sample of 28 participants enrolled in the parent trial by May 2025 with usable whole body DXA and HR-pQCT scans of the lower leg was selected to assess the relationship between anatomically matched, regional leg muscle metrics derived from DXA and HR-pQCT, obtained from regions corresponding to the 30% offset site of the distal tibia on the non-dominant leg (defined as the leg the participant would not use to kick a ball) (Figure 1). Participants were recruited from the Atrium Health Wake Forest Baptist Weight Management Center in Winston-Salem (*N* = 25), NC and the Atrium Health Weight Management Center in Charlotte, NC (*N* = 3) as part of the main trial. Written informed consent was obtained from all participants. The study was approved by the Wake Forest University Institutional Review Board (IRB00074763).

**Figure 1:**
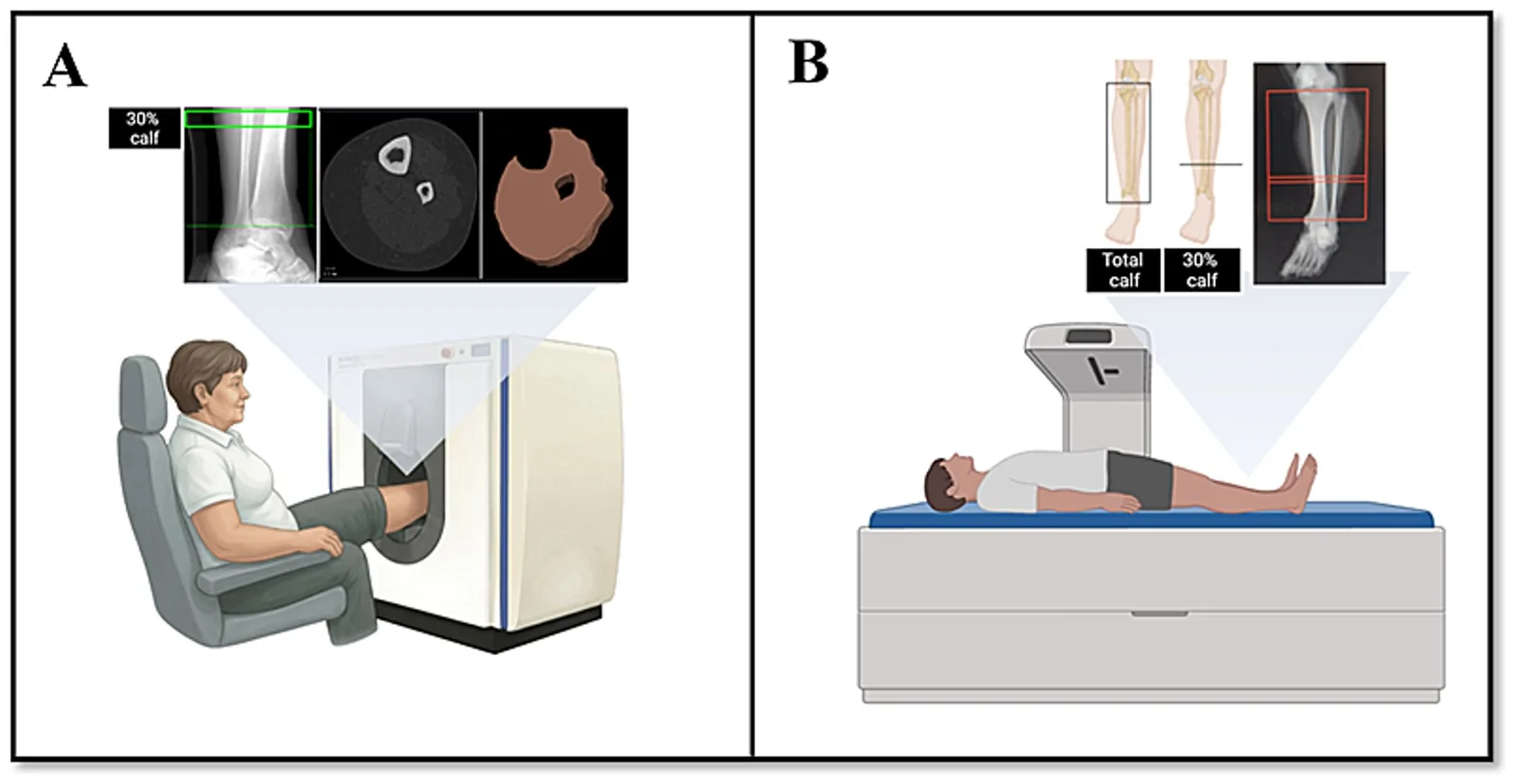
Assessment of calf muscle measures using HR-pQCT and DXA. (A) HR-pQCT scan of the lower leg acquired at the 30% offset site of the distal tibia, illustrating positioning, axial imaging, and muscle segmentation used to derive muscle CSA and density. Panel A was created using AI-based illustration tools (https://illustrae.co/). (B) Whole-body DXA scan illustrating the total calf region and the anatomically matched 30% calf subregion used to extract calf lean mass from the same leg and anatomical location as the HR-pQCT scan. Panel B was created with BioRender.com. CSA, cross-sectional area.

### Procedures

#### Demographics

Baseline demographic characteristics, including age, sex, and race, were collected via self-report.

#### Anthropometry

Height and body weight were measured using a calibrated stadiometer and scale (TANITA #WB-3000PLUS, TANITA Corp).

#### HR-pQCT

HR-pQCT (XtremeCT II, Scanco Medical AG) was used to assess leg muscle CSA (cm^2^) and muscle density (average Hounsfield Unit, HU) at the 30% leg site of the non-dominant leg (Figure 1A), unless fracture history required scanning the dominant leg (*n* = 1). Shank length was assessed by soft measuring tape, and the 30% site defined as the distance from the distal medial malleolus to the medial tibial plateau with the subject sitting in the chair, and foot flat on the floor. A standard scanning protocol was used with a nominal isotropic voxel size of 60.7 μm, tube voltage of 68 kVp, tube current of 1470 mA, and an integration time of 43 ms. For each scan, a total of 168 slices were acquired, resulting in a total stack length of 10.2 mm. All scans were carefully examined for motion artifacts, and the quality was graded from 1 (no motion) to 5 (significant blurring) based on a visual grading system.^31^ If the quality was 3-5, up to 2 additional scans were performed. For muscle tissue analysis, all 168 slices were exported from the scanner and downsampled by a factor of 3 as has been done previously to reduces computational complexity and the overall segmentation workload while retaining sufficient spatial resolution for muscle tissue analysis,^32^ resulting in an isotropic voxel size of 0.1821 mm. The downsampled images were analyzed using a custom Python script (Python v3.13, Python Software Foundation) implementing a custom, internally trained and maintained nnU-Net predictive model for aiding muscle segmentation (v2.6.2).^33^ Briefly, the U-Net self-configuring architecture has been trained in an iterative manner, starting with small sample sizes that are used to bootstrap (or expand the size of) the training cohort for future iterations. Each iteration of training was checked and manually corrected prior to the next round of training commencing. The training sample size at the time of this study was *n* = 39 total subjects, including both radius (*n* = 19) and tibia (*n* = 20) HR-pQCT scans obtained in our laboratory. One of these tibia scans overlapped with participants included in the present study, while the remaining training scans were derived from independent datasets. Training data included both fat and bone segmentations alongside muscle, as internal testing showed increased accuracy of the muscle boundary when including other tissues. Further details regarding the U-Net setup, training and validation will be made available in future work. This U-Net was applied to all 28 individuals for this pilot study, and the segmentation outputs were manually corrected prior to extraction of HR-pQCT muscle characteristics (J.R.S.). Manual corrections primarily involved removal of non-muscle tissue incorrectly included in the segmentation (eg, adjacent to the tibia) and minor adjustments to ensure consistent delineation of muscle boundaries. To evaluate reproducibility, muscle CSA from the automated segmentation was compared with manual segmentations in a subsample (*n* = 12). The mean difference was small and not statistically significant (0.03 cm^2^, *p* = .83). Measurement error was low (CV = 2.5%) and Bland–Altman analysis showed minimal bias (0.08 cm^2^) with 95% limits of agreement from −2.44 to 2.60 cm^2^. Following quality analysis of muscle segmentations, an additional Python script implemented muscle characteristic extraction using the NIH funded SimpleITK (v2.5.2) to apply basic physical analyses of volume calculation (Equation 1) and average density extraction over all pixel values in the segmentation.^34^ Muscle CSA was subsequently derived from muscle volume according to Equation 2.


(1) \begin{equation*} \mathrm{Muscle}\ \mathrm{volume}=\mathrm{Pixel}\ \mathrm{spacing}\,\ast \mathrm{Total}\ \mathrm{number}\ \mathrm{of}\ \mathrm{pixels} \end{equation*}

(2) \begin{equation*} \mathrm{Cross}\ \mathrm{sectional}\ \mathrm{area}=\frac{\mathrm{Muscle}\ \mathrm{volume}}{\mathrm{Pixel}\ \mathrm{spacing}\ast \mathrm{number}\ \mathrm{of}\ \mathrm{slices}} \end{equation*}

#### DXA

Whole-body composition was evaluated using whole-body DXA (Hologic Horizon A) to estimate LBM, ALM, fat mass, and fat percentage. When necessary due to body size, a standard half-body scanning procedure was used, with the contralateral side mirrored by the software according to the manufacturer’s protocol. Subsequently, scans were reanalyzed using Hologic software (Hologic APEX Software version 5.6.1.3 rev 006) to derive lean mass (kg) for the subregion corresponding to the 30% offset site of the same leg that was scanned with HR-pQCT, ensuring anatomical matching between imaging modalities (Figure 1). Detailed procedures for defining the leg subregion are provided in the Supplementary Material 1. Briefly, identification of the 30% offset leg subregion was performed in 2 sequential steps. First, the total leg region was defined as the area between the knee and ankle joints, identified by the proximal end of the tibia and the distal end of the tibia, respectively, and the “leg lean mass” measure was output. Second, the 30% offset site was determined by measuring 30% of the distance from the distal tibia (ankle joint) toward the knee joint. At this level, a subregion box was placed with the minimum height allowed by the software (corresponding to approximately 2-3 cm) and lean mass in this subregion was output as the “30%-leg lean mass” measure. Given the manual procedure required to define the 30% offset subregion, the reliability of the placement process was assessed prior to data collection. Two assessors performed the subregion placements (S.G. and V.J.), and test–retest analyses showed no systematic changes in performance. The pooled intraclass correlation coefficient (ICC) was 1.00 (95% CI: 0.99-1.00), with a SEM of 0.0151 kg and SEM% of 0.7% for the total leg, and ICC was 0.96 (95% CI: 0.93-0.98), with a SEM of 0.0062 kg and SEM% of 5.5% for the 30% offset leg. Both assessors demonstrated comparable reliability metrics.

#### Gait speed

Gait speed (m/s) was assessed using the fast 400-m walk test.^35^ Participants were instructed to walk back and forth between 2 cones placed 20 m apart in an indoor hallway until completing a total distance of 400 m. Total time was recorded, and gait speed was calculated as total distance divided by time.

#### Lower limb muscle power

Lower limb muscle power was assessed using the Stair Climb Power Test. Participants ascended a 12-step staircase (step height 17.8 cm, total vertical distance 2.1 m) as fast as possible on 2 occasions, with a 1-min rest between trials. The fastest attempt was used for analysis. Muscle power (W) was calculated as potential energy change over time^36^:


$$ \mathrm{Power}=\frac{\Delta{E}_{\mathrm{pot}}}{\Delta t}=\frac{\mathrm{body}\ \mathrm{mass}\times g\times h}{\mathrm{time}} $$

where *g* = 9.81 m/s^2^ and *h* = 2.1 m. The test has demonstrated excellent intersession reliability (ICC = 0.93-0.95) for time, power (W), and power/body weight (W·kg^−1^) in adults with obesity.^37^

#### Muscle strength

Isokinetic knee extensor strength of the non-dominant leg, which was also the leg used for HR-pQCT and DXA-derived leg muscle measurements, was assessed using an isokinetic dynamometer (Humac Norm, CSMi). Participants performed 5 maximal contractions at an angular velocity of 60°/s, with 1-min rest intervals between trials. The highest torque value (Nm) and torque/body weight (Nm·kg^−1^) were used for analysis. Previous studies have shown excellent intersession reliability for knee extension at 60°/s in adults with obesity (ICC = 0.95).^38^

### Statistics

All data are presented as mean and SD. Prior to statistical analysis, outcomes were tested for normality using quantile–quantile (Q–Q) plots. For the main analysis, Pearson correlation coefficients and simple linear regression analyses were used to assess the relationship between HR-pQCT-derived muscle CSA and density and DXA-derived lean mass at the total leg and 30% offset site. The strength of the correlations was interpreted according to the following classification: <0.1 (negligible), 0.1-0.39 (weak), 0.4-0.69 (moderate), 0.7-0.89 (strong), and ≥0.9 (very strong).^39^ A similar approach was used to examine the relationships between HR-pQCT and DXA imaging metrics (eg, HR-pQCT muscle CSA, DXA lean mass) and measures of isokinetic knee strength, lower limb muscle power, and gait speed. The significance level was set at *p* ≤ .05 (2-tailed). All analyses were performed using Stata statistical software, version 18 (StataCorp) and GraphPad Prism, version 10 (GraphPad Software).

## Results

### Participant characteristics

The study included 28 adults with class 3 obesity (27 female, 1 male) with a mean ± SD age of 47.7 ± 8.6 yr. Participants had a mean body weight of 120.9 ± 18.2 kg and BMI of 43.8 ± 4.6 kg/m^2^. Additional body composition, leg muscle, and functional measures are presented in Table 1. Complete data were not available for all muscle function tests. Missing values occurred due to time constraints, elevated blood pressure at the assessment visit, and weight exceeding equipment limits. Consequently, isokinetic knee strength was available for 24 participants, and gait speed and lower-limb muscle power were available for 26 participants.

**Table 1:** Participant characteristics (*n* = 28).

**Age (yr)**	**47.7 ± 8.6 [31.9-63.6]**
**Female, *n* (%)**	27 [96%]
**Race, *n* (%)**	
**White/Caucasian**	18 (64%)
**Black or African American**	10 (36%)
**Body weight (kg)**	120.9 ± 18.2 [91.9-173.3]
**Height (cm)**	165.8 ± 6.7 [154.0-84.8]
**BMI (kg/m** ^**2**^ **)**	43.8 ± 4.6 [36.5-55.9]
**DXA acquired metrics**	
**Total body fat (%)**	46.8 ± 3.9 [36.8-55.9]
**Total fat mass (kg)**	62.5 ± 10.0 [33.8-83.1]
**Total LBM (kg)**	63.4 ± 8.3 [46.0-83.1]
**ALM (kg)**	27.4 ± 3.3 [19.6-33.7]
**Total leg lean mass (kg)**	2.23 ± 0.40 [1.5-3.3]
**30% leg lean mass (kg)**	0.107 ± 0.019 [0.074-0.148]
**HR-pQCT acquired metrics**	
**Leg muscle CSA (cm**^**2**^**)**	36.0 ± 4.8 [28.7-50.3]
**Leg muscle density (HU)**	60.1 ± 8.1 [44.3-88.0]
**Muscle function and strength measures**	
**Isokinetic peak torque (Nm)^a^**	111.9 ± 30.1 [57.7-170.5]
**Relative isokinetic peak torque (Nm/kg body weight)^a^**	1.0 ± 0.2 [0.5-1.4]
**Lower limb muscle power (W)^b^**	382.2 ± 96.9 [159.4-590.9]
**Lower limb muscle relative power (W/kg body weight)^b^**	3.2 ± 0.6 [1.6-4.1]
**400-m average gait speed (m/s)^b^**	1.1 ± 0.1 [0.7-1.4]

Demographic, anthropometric, body composition, leg muscle, and muscle function characteristics for the participants are presented as means ± SD or *n* (%). Brackets [] indicates range. Abbreviations: ALM, appendicular lean mass; CSA, cross-sectional area; LBM, lean body mass.

a Measures were available for *N* = 24 participants.

b Measures were available for *N* = 26 participants.

### Associations between DXA-derived lean mass and HR-pQCT muscle metrics

Mean values and standard deviations for DXA-derived lean mass and HR-pQCT-derived muscle metrics are shown in Table 1. Correlations between DXA and HR-pQCT measures are presented in Figure 2. For total leg measures, DXA-derived leg lean mass showed a moderate positive correlation with HR-pQCT muscle CSA (*r* = 0.57, *p* = .0017), whereas the correlation with HR-pQCT muscle density was negligible (*r* = 0.04, *p* = .84). At the 30% leg site, DXA-derived leg lean mass also demonstrated a moderate positive correlation with HR-pQCT muscle CSA (*r* = 0.69, *p* < .0001), while the correlation with HR-pQCT muscle density was again negligible (*r* = −0.01, *p* = .96). In a sensitivity analysis excluding 2 potential outliers identified based on visual inspection of the scatterplots, the strength of the association between DXA-derived leg lean mass and HR-pQCT muscle CSA increased for both total leg (*r* = 0.71) and the 30% leg site (*r* = 0.79). In contrast, associations between HR-pQCT muscle CSA and larger DXA-derived regions were weaker, including body weight (*r* = 0.33, *p* = .084), total lean mass (*r* = 0.32, *p* = .093), and appendicular lean mass (*r* = 0.43, *p* = .023). Correlations within DXA-derived lean mass measures showed strong associations between total leg LBM, LBM, and ALM (*r* = 0.70-0.75, all *p* < .001), whereas the 30% leg region was less strongly associated with LBM (*r* = 0.33, *p* = .081) and ALM (*r* = 0.50, *p* = .007). LBM and ALM were highly correlated (*r* = 0.90, *p* < .0001).

**Figure 2:**
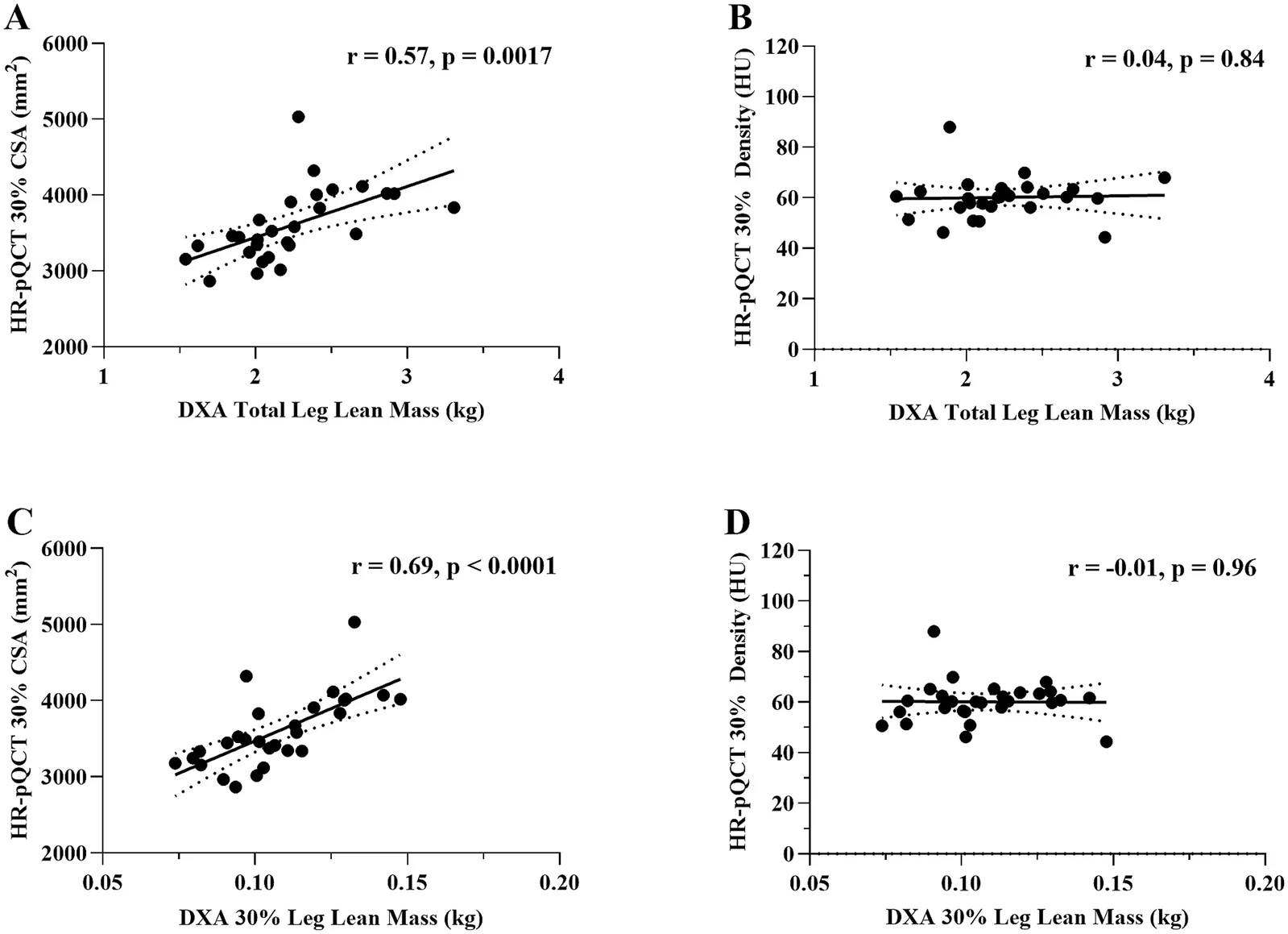
Associations between DXA-derived calf lean mass and HR-pQCT-derived calf muscle metrics. Panels A and B show the relationships between DXA-derived total calf lean mass and HR-pQCT muscle cross sectional area (A) and muscle density (B). Panels (C) and (D) show the corresponding relationships using DXA derived lean mass from the 30% calf region compared with HR-pQCT muscle CSA (C) and muscle density (D). Solid lines represent linear regression fits, and dotted lines indicate the 95% confidence bands. Pearson correlation coefficients (*r*) and *p*-values are displayed in each panel. CSA, cross-sectional area.

### Relationship of DXA and HR-pQCT measures with muscle function and physical performance

Correlations between body composition, muscle metrics, and measures of muscle function and physical performance are presented in Table 2. Significant correlations were observed primarily for absolute muscle power which was positively associated with body weight (*r* = 0.70, *p* < .001), total LBM (*r* = 0.63, *p* < .0001), appendicular lean mass (*r* = 0.42, *p* = .033), and total leg lean mass (*r* = 0.40, *p* = .042). For muscle strength, HR-pQCT leg muscle CSA showed a moderate positive association with knee extensor peak torque (*r* = 0.41, *p* = .048). No other significant correlations were observed between the imaging-derived muscle metrics and functional outcomes, including relative torque, relative muscle power, or gait speed.

**Table 2:** Pearson correlations between muscle metrics, physical function, and muscle strength.

	**Knee extension peak torque (Nm)**		**Knee extension peak torque (Nm/kg)**		**Muscle power (W)**		**Muscle power (W/kg)**		**Gait speed (m/s)**	
	***r***	***p***	***r***	***p***	***r***	***p***	***r***	***p***	***r***	***p***
**Body weight (kg)**	0.34	.100	−0.14	.514	**0.70**	**<.001**	0.14	.484	0.03	.892
**DXA**										
** Total LBM (kg)**	0.24	.257	−0.17	.438	**0.63**	**<.001**	0.13	.527	0.02	.917
** ALM (kg)**	0.20	.340	−0.15	.494	**0.42**	**.033**	−0.06	.773	−0.19	.360
** Total leg lean mass (kg)**	0.23	.276	−0.09	.662	**0.40**	**.042**	−0.03	.871	−0.13	.529
** 30% leg lean mass (kg)**	0.31	.144	0.16	.450	0.17	.396	−0.03	.901	−0.12	.568
**HR-pQCT**										
** Leg muscle CSA (cm** ^**2**^ **)**	**0.41**	**.048**	0.24	.268	0.19	.346	−0.01	.963	−0.01	.950
** Leg muscle density (HU)**	−0.12	.585	−0.02	.937	−0.08	.692	−0.05	.824	0.17	.403

Data are presented as Pearson correlation coefficients (*r*). Bold values indicate statistically significant correlations (*p* < 0.05). Abbreviations: ALM, appendicular lean mass; CSA, cross-sectional area; LBM, lean body mass.

## Discussion

The purpose of this cross-sectional pilot study was to examine the association between DXA- and HR-pQCT-derived leg muscle measures and to evaluate how these imaging-derived muscle metrics relate to muscle strength and physical function in adults with obesity. The main findings were that HR-pQCT-derived leg muscle CSA showed moderate positive correlations with DXA-derived total leg lean mass (*r* = 0.57) and with lean mass from the anatomically matched 30% leg site (*r* = 0.69), whereas HR-pQCT-derived leg muscle density was not related to DXA lean mass at either level. Associations were weaker when larger DXA-derived regions were examined, such as LBM and ALM, suggesting that region-specific comparisons between DXA and HR-pQCT improve the observed associations. This may indicate that local HR-pQCT-derived muscle CSA reflects regional muscle morphology rather than overall lean mass status. Regarding muscle function, DXA-derived lean mass measures (total body, appendicular, and total leg) were positively associated with absolute lower limb muscle power, while HR-pQCT leg muscle CSA was moderately correlated with absolute knee extensor peak torque; neither modality showed meaningful associations with relative strength, relative muscle power, or gait speed. Taken together, these findings partially support our hypothesis, confirming positive associations between HR-pQCT muscle CSA and DXA lean mass, while associations with muscle density and functional outcomes were more limited.

In this study, moderate positive correlations were observed between HR-pQCT-derived muscle CSA and DXA-derived lean mass at both the total leg and the 30% leg site, with slightly stronger correlations when anatomical regions were closely matched at the 30% site. Prior studies indicate that DXA-derived lean mass strongly associates with CT- (*R*^2^ ≈ 0.96) and MRI-based (*r* ≈ 0.95) muscle measures at the whole-leg level,^18^^,^^40^^,^^41^ whereas associations weaken as assessments become more localized (*r* ≈ 0.86-0.91).^40^^,^^41^ Similarly, in adults with overweight or obesity, CT-derived muscle CSA correlates strongly with DXA-derived total and appendicular lean mass, whereas associations with muscle density are negligible.^13^ DXA-derived lean mass has also been compared with pQCT, where moderate associations have been observed between DXA-derived leg lean mass and pQCT-derived leg muscle CSA (*R*^2^ = 0.48-0.49), whereas associations with muscle density were negligible (*R*^2^ = 0.02-0.03).^42^ First- and second-generation HR-pQCT have also been compared with pQCT, demonstrating very strong agreement for muscle CSA (*R*^2^ ≈ 0.95) and more moderate agreement for muscle density (*R*^2^ ≈ 0.66).^21^ Overall, these findings indicate that DXA aligns closely with CT and MRI for whole-limb muscle assessment, whereas associations with peripheral imaging modalities such as pQCT and HR-pQCT appears more modest, while peripheral imaging techniques demonstrate stronger agreement with each other. The stronger associations observed between DXA-derived lean mass and muscle CSA, compared with muscle density, are expected given that DXA primarily reflects muscle quantity, whereas muscle density captures tissue composition and intramuscular fat infiltration. As DXA cannot distinguish muscle from non-contractile components within lean tissue, weak associations with muscle density are biologically and methodologically plausible.

### Associations between muscle measures and functional outcomes

In the present study, body weight and DXA-derived lean mass measures were not associated with knee extensor strength or gait speed, but were positively associated with absolute stair climb power. The strength of these associations decreased as lean mass measures became more region-specific (total body > appendicular > total leg > 30% leg) (*r* = 0.17-0.70), suggesting that the associations with absolute stair climb power may be partly driven by overall body mass rather than muscle quantity per se. This finding was somewhat surprising, as isokinetic knee extensor strength has previously been shown to correlate with both total LBM and appendicular lean mass in larger cohorts of older adults (*r* ≈ 0.30-0.55).^28^^,^^43^ Related unpublished data from the ZABAS study in adults with obesity undergoing bariatric surgery demonstrated strong positive correlations between absolute measures of isokinetic muscle strength and stair climb muscle power and both total and appendicular lean mass at baseline and at 12 mo post-surgery (*r* = 0.70-0.88, *p* < .0001), whereas correlations between body weight and absolute muscle strength and power were only moderate (*r* = 0.57-0.68, *p* < .0001).^5^ These discrepancies may partly be explained by methodological and contextual factors. DXA provides stable measures of LBM with low measurement error (CV ≈ 1.6%),^16^^,^^17^ whereas tests of muscle power and isokinetic muscle strength depend on maximal voluntary performance and exhibit substantially greater variability in adults with obesity (CV ≈ 6%-7%).^37^^,^^38^ Consequently, measured performance may be influenced by day-to-day fluctuations, reduced effort, pain, fatigue, or comorbidities and may not always reflect true maximal capacity. In relatively small samples, correlation estimates are therefore particularly sensitive to submaximal test performance, such that limited effort in a few participants can substantially attenuate observed associations. Additionally, methodological differences in test execution across studies, including instructions, familiarization, and tester-related factors, may further contribute to variability in the relationships between DXA-derived muscle measures and functional outcomes.

For the HR-pQCT-derived muscle metrics, we found no associations between leg muscle density (HU) and any of the functional or strength outcomes. Leg muscle CSA was moderately correlated with knee extensor peak torque (*r* = 0.41), but showed no relationships with relative strength, muscle power, or gait speed. In contrast, Warden et al. reported, using multivariable regression models in a large reference cohort of community-dwelling adults, that HR-pQCT-derived muscle density independently predicted grip strength (*R*^2^ = 0.65), fast gait speed (*R*^2^ = 0.23), and self-reported physical function (*R*^2^ = 0.37) after adjustment for ALM/height^2^, BMI, and whole-body percent fat, whereas muscle density was not associated with chair-rise performance.^29^ Similarly, Chow et al. examined HR-pQCT-derived muscle density and muscle volume at the distal tibia in adult women and reported that muscle density was associated with both lower-limb muscle strength and chair-rise performance, whereas muscle volume, which can be directly converted to CSA by normalizing to scan length, was not associated with muscle strength (*r* = 0.25) but was moderately associated with chair-rise performance (*r* = −0.48).^44^ Taken together, findings across studies are inconsistent, likely reflecting differences in how HR-pQCT muscle metrics and functional outcomes are defined, analyzed, and reported, as well as variation in strength and functional testing approaches. In the present study, DXA-derived leg lean mass was also not associated with knee extensor strength, suggesting that leg-based muscle measures, regardless of imaging modality, may have limited utility for predicting strength in larger, non-local muscle groups such as the quadriceps. However, these conclusions are based on a relatively small sample, and it cannot be excluded that a larger study would reveal more robust or consistent associations,^30^ warranting cautious interpretation of the present findings.

### Methodological considerations and implications for research and practice

Although we observed the strongest correlations for anatomically matched regions, DXA-derived leg lean mass and HR-pQCT leg muscle CSA were only moderately correlated. Our findings also suggest that the strength of the association decreases when larger DXA-derived regions are used, indicating that region-specific comparisons between imaging modalities may be important when evaluating localized muscle morphology. This supports the notion that HR-pQCT-derived CSA primarily reflects regional muscle characteristics rather than overall lean mass status. This interpretation is supported by the within-DXA analyses, where strong associations were observed between global measures (eg, LBM and ALM), while more localized measures, such as the 30% leg region, showed weaker correlations with these global indices, suggesting that regional DXA measures are not simply scaled representations of whole-body lean mass. The lack of perfect association may be related to fundamental differences in measurement principles, as DXA provides a 2D estimate of lean soft tissue that includes both muscle and non-contractile components, whereas HR-pQCT directly quantifies 3D muscle CSA based on tissue attenuation and segmentation. In addition, small discrepancies in region definition and partial volume effects, particularly in individuals with obesity, may further contribute to measurement variability even when anatomical sites are closely matched. Another contributing factor may be the manual placement of DXA regions of interest, which may weaken the association between the 2 imaging modalities. Although assessor reliability was high (ICC = 0.96) with a relatively low standard error of measurement (SEM ≈ 5.5%), even small misplacements of the DXA region of interest by 1 to 2 pixels have been shown to result in differences of up to 10%-20% in lean mass estimates. To mitigate this source of variability, DXA leg subregion analyses were performed twice, and the mean value was used in the analyses. Overall, DXA-derived leg lean mass represents a pragmatic and time-efficient approach to regional muscle assessment, as both leg and mid-thigh subregion analyses can be completed within minutes using existing whole-body DXA scans. Although association with HR-pQCT is only moderate, careful region placement and averaging of repeated analyses can improve robustness, supporting the use of DXA-based regional muscle measures in large-scale or clinical studies where more advanced imaging is not feasible.

### Strength and limitations

A key strength of this study is the anatomical matching between modalities, as HR-pQCT scans were acquired at the 30% tibial length site and DXA subregions were defined to correspond to the same leg and anatomical level. We also extracted 2 DXA-derived leg regions (total leg and the anatomically matched 30% leg site), enabling evaluation of associations across both broader and more localized regions. The study was conducted in a clinically relevant population of adults with obesity eligible for bariatric surgery. Finally, muscle function outcomes were assessed using tests with documented reliability in adults with obesity, supporting confidence in the functional measures.

This pilot study has several limitations. The small sample size limits statistical power and makes correlation estimates more sensitive to variability in test performance. The cross-sectional design prevents evaluation of longitudinal change or responsiveness of the imaging-derived muscle measures. In addition, the definition of the 30% leg region using DXA relies on manual ROI placement and may be influenced by participant repositioning between repeated whole-body scans. While ROI placement showed high assessor reliability, scan–rescan precision of the complete DXA measurement procedure was not evaluated. This should be established in future methodological studies, particularly before applying this approach in longitudinal intervention studies. In addition, the cohort was predominantly female, which limits generalizability, particularly to men with obesity. Furthermore, ankle plantarflexor strength was not assessed. While knee extension strength was included as a general measure of lower-limb muscle function in the parent trial, plantarflexion strength would have provided a more anatomically specific functional measure for the leg musculature.

### Conclusion

HR-pQCT-derived leg muscle CSA was moderately correlated with DXA-derived leg lean mass, whereas muscle density was not. Correlations between leg muscle measures and muscle strength or physical performance were generally weak and inconsistent. Overall, these findings indicate limited associations between leg-based imaging measures and functional outcomes in adults with obesity.

## Supplementary Material

260421_SOP_Dual-energy_X-ray_Absorptiometry_final_ziag158

## Data Availability

The data supporting the findings of this study are not publicly available due to their association with an ongoing clinical trial. Requests for access to the data will be considered on a case-by-case basis and subject to relevant approval.
