# Supplementary material for: Leg muscle assessment by HR-pQCT and DXA: associations between imaging measures and muscle function in adults with obesity—a pilot study
Source: JBMR Plus. 2026 Sep 22;10(11):ziag158. doi: 10.1093/jbmrpl/ziag158 (PMC13644820; doi:10.1093/jbmrpl/ziag158)
Supplement: Supplementary file 1 [file 260421_sop_dual-energy_x-ray_absorptiometry_final_ziag158.docx]

**Standard Operating Procedure (SOP) Dual-energy X-ray Absorptiometry (DXA) for Subregion Analysis for Lower Leg**

Version: 1.0
Date: [06/27/2024]
Prepared by: [Søren Gam, PhD Candidate, MSc Sport and Health Science]
Approved by: [Kristen M. Beaver, Associated Professor]

Table contents

[1.0 Revision History	1](#_Toc227654998)

[2.0 Purpose	2](#_Toc227654999)

[3.0 Scope	2](#_Toc227655000)

[4.0 Introduction	2](#_Toc227655001)

[5.0 Body Composition Analysis	2](#_Toc227655002)

[6.0 Materials	2](#_Toc227655003)

[7.0 Procedures	3](#_Toc227655004)

[7.1 Select the scan for analysis.	3](#_Toc227655005)

[7.2 Subregion Placement	4](#_Toc227655006)

[7.3 Total lower leg, 50% lower leg, and 30% lower leg	4](#_Toc227655007)

[7.3.1 Total lower leg	4](#_Toc227655008)

[7.3.2 Mid-leg and 30% Leg (straight boxes)	5](#_Toc227655009)

[7.5 Results	5](#_Toc227655010)

[8.0 Quality Control and Assurance	5](#_Toc227655011)

[8.2 Assessor reliability	6](#_Toc227655012)

[8.2.3 Study design and statistics	6](#_Toc227655013)

[8.2.3 Reliability for length measurements	7](#_Toc227655014)

[8.2.4 Reliability for Lean body mass for the subregion	7](#_Toc227655015)

# 1.0 Revision History

| **Date** | **Version** | **Description of changes** | **Author** | **Approved by** |
| --- | --- | --- | --- | --- |
|  |  |  |  |  |
|  |  |  |  |  |
|  |  |  |  |  |
|  |  |  |  |  |
|  |  |  |  |  |
|  |  |  |  |  |
|  |  |  |  |  |

# 2.0 Purpose

The purpose of this Standard Operating Procedure (SOP) is to provide detailed instructions for performing dual-energy X-ray absorptiometry (DXA) analysis specifically for muscle measurement. This SOP aims to ensure accuracy, consistency, and reliability in the data collected during DXA procedures related to muscle mass and composition. By following these guidelines, users can achieve standardized results that are essential for medical diagnostics, research, and health assessments of muscle health.

# 3.0 Scope

This SOP applies to all personnel involved in the DXA analysis process within the Department of Health and Exercise Science at Wake Forest University, for the purpose of measuring muscle mass and composition. It covers the procedures for preparing and conducting muscle analysis DXA whole-body scans, placing subregion boxes for detailed muscle analysis, and ensuring the quality and accuracy of the measurements. The scope includes:

- Selection and preparation of scans for muscle analysis.
- Detailed steps for placing subregions on the lower leg to measure lean body mass.
- Measurement and analysis of lean body mass and other relevant metrics.
- Quality control measures to ensure data reliability.
- Training requirements for new assessors to achieve consistent results.

This SOP is intended for use by technicians, researchers, and healthcare professionals who are responsible for conducting and analyzing DXA scans for muscle measurement. It provides the necessary guidelines to maintain high standards of practice and data integrity in muscle-related DXA analysis.

# 4.0 Introduction

Dual-energy X-ray absorptiometry (DXA) is a highly accurate and widely used technique for measuring bone mineral density and body composition. It provides detailed insights into fat mass, lean mass, and bone density, making it an essential tool in medical diagnostics, research, and health assessments. The precision and reliability of DXA contribute significantly to the evaluation and monitoring of various health conditions and interventions.

# 5.0 Body Composition Analysis

Hologic’s Body Composition Analysis provides the ability to analyze the soft tissue composition of the entire body and to follow the changes in soft tissue composition over time in response to interventions such as diet and exercise. Analysis of fat mass, lean mass, and % fat mass can be reported for the entire body, head, arms, trunk, pelvis, and legs. Further analysis of specific subregions can be performed, and the following sections will explain how to do this. This SOP is based on Hologic APEX Software version 5.6.1.3 rev 006.

# 6.0 Materials

- Software: Hologic APEX Software version 5.6.1.3 rev
- Physical ruler
- Pen and paper for noting length
- Calculator

# 7.0 Procedures

## 7.1 Select the scan for analysis.

1. Select the "**Analyze Scan**" option in the main menu. See picture 1

.

1

2. Select "**All Scans**". See picture 2

2

3. Find and select the participants of interest and choose “**Whole Body**”. See picture 3.

3

## 7.2 Subregion Placement

4

In the following section, a description is provided on how to place the subregions for the lower leg, and 50% and 30% lower leg offset. Depending on the purpose, it may not be necessary to place all subregions.

1. "**Subregions**" will become visible when the tools "1) **Regions**" and "2) **A/G Regions**" have been selected. Select the tool "3) **Subregions**. See picture 4.

2. In the tool "**Subregions**" you can add and modify the placed subregions. Subregions can be added when "**Whole Mode**" is selected. The **+ sign** will add a new subregion, and the **- sign** will delete the selected subregion.

3. The buttons << and >> in "Select a region" shift between added subregions and can be useful. The mouse can also be used for shifting or selecting an already placed subregion, but there is a risk of rotating or dragging the subregion.

4. Moving Subregions: Both the mouse and arrows on the keyboard can be used to move the selected subregion.

5. In Line Mode, the subregion size can be modified. Yellow dashed lines indicate which side is selected.

6. Point Mode can make additional adjustments but is not useful for the purpose of this SOP.

## 7.3 Total lower leg, 50% lower leg, and 30% lower leg

Subregions for the total leg are defined by placing subregion boxes along a horizontal line between **the knee joint** and **ankle joint**, respectively, see picture 5. The ankle joint is defined by the distal end of the tibia. Mid-leg and 30% offset leg are defined as 50% and 30% offsets from the ankle joint towards the knee joint, see picture 8.

5

### 7.3.1 Total lower leg

1. In **Whole Mode**, add a subregion and drag/move it so the top line of the subregion touches the midline through the knee joint.

2. In **Line Mode**, elongate the subregion length by selecting the bottom line of the subregion and increase the length by using the ↓ key or by dragging with the mouse until the line is just below the distal part of the tibia.

3. In **Line Mode**, define the width of the subregion by respectively selecting the medial and lateral lines and increasing them until the whole leg is included.

### 7.3.2 Mid-leg and 30% Leg (straight boxes)

1. Use a **physical ruler** to measure the distance from the top to the bottom of the subregion box by placing the ruler directly on the computer monitor. Be as accurate as possible and try to keep your head in the same position every time, as this will influence how you read the length.

6

2. In **Whole Mode**, add a new subregion and shrink the height to the smallest possible height. Note: The size of the new subregion will be the same as the previously selected subregion.

3. Drag the new subregion to the position that corresponds to e.g. 50% and 30% offset from the distal tibia towards the knee joint. While doing this, place the ruler on the screen and use the up and down arrow keys to adjust the placement. See picture 6

4. When all subregion boxes are placed, press on the bottom “**Subregion results**” for saving

7.5 Results
In the results file, on the page for whole-body composition, you will find the results for the defined subregions. In this case, we have defined three subregions: total leg (R1), 50% leg (R2), and 30% leg (R3). See picture 7.

7

8.0 Quality Control and Assurance

Ensuring the accuracy and reliability of DXA measurements is critical for valid muscle mass and composition analysis. This section outlines the procedures and metrics used to assess the consistency and precision of measurements, including the comparison between physical and built-in software rulers, as well as the reliability of assessor performance over time.

8.1 Physical ruler and software ruler

To determine how the physical and built-in software rulers correspond, two distances for the leg (total length and 30% offset) for 21 persons were measured using a physical ruler and the built-in software ruler, showing a very strong association with R² = 0.99.

To transform lengths measured by the physical ruler to DXA-determined lengths, and thereby determine the length of, for example, the thigh and leg, a linear regression analysis was performed to derive an equation for converting lengths:

DXA length (cm) = 8.9558 * ruler length (cm) + 0.2599

Two control lengths were subsequently measured to check the validity of the equation, revealing a small deviation of 0.2 mm between the predicted and DXA-derived lengths. A deviation of 0.2 mm corresponds to one click up or down. In Table 1, corresponding values for the physical and built-in ruler measurements are displayed.

In conclusion, the ruler length measured directly on the monitor corresponded closely with the built-in ruler in the software.

| **Table 1:** Physical ruler and corresponding values in cm | | | | | |
| --- | --- | --- | --- | --- | --- |
| **Physical** | **DXA ruler** | **Physical** | **DXA ruler** | **Physical** | **DXA ruler** |
| 3.5 | 31.6 | 4.5 | 40.6 | 5.5 | 49.5 |
| 3.6 | 32.5 | 4.6 | 41.5 | 5.6 | 50.4 |
| 3.7 | 33.4 | 4.7 | 42.4 | 5.7 | 51.3 |
| 3.8 | 34.3 | 4.8 | 43.2 | 5.8 | 52.2 |
| 3.9 | 35.2 | 4.9 | 44.1 | 5.9 | 53.1 |
| 4 | 36.1 | 5 | 45.0 | 6 | 54.0 |
| 4.1 | 37.0 | 5.1 | 45.9 | 6.1 | 54.9 |
| 4.2 | 37.9 | 5.2 | 46.8 | 6.2 | 55.8 |
| 4.3 | 38.8 | 5.3 | 47.7 | 6.3 | 56.7 |
| 4.4 | 39.7 | 5.4 | 48.6 | 6.4 | 57.6 |

## 8.2 Assessor reliability

### 8.2.3 Study design and statistics

Muscle metrics from 15 participants (11 baseline and 4 six-month follow-ups) were collected three times to assess drift in performance and the reliability of the assessor. Test 1 and Test 2 were collected on separate days to indicate between-day reliability, while Test 2 and Test 3 were collected on the same day to indicate within-day reliability.

To assess systematic bias between tests, a repeated measures ANOVA was applied. In the case of a significant difference, a Tukey post hoc test was used to identify where the difference was present. Intraclass correlations (ICC1.1) with 95% confidence intervals (95% CI) were used to evaluate the agreement between successive measurements. The ICC values were classified as follows: <0.5 (poor), 0.5−0.75 (moderate), 0.75−0.90 (good), and >0.90 (excellent) (Koo & Li, 2016). The within-subject coefficient of variation (CV_W-S)_ was calculated to further evaluate test reproducibility. CV_W-S_ was defined as the standard deviation (SD) divided by the grand mean, where SD was derived using the formula: SD = √(∑(d²)/2n), where d is the test−retest difference (Holsgaard Larsen et al., 2007).

Measurement error was presented as the standard error of measurement (SEM) and the SEM%. SEM was calculated using the formula: SD × √(1−ICC) (Hopkins, 2000; Weir, 2005). SEM% was calculated by dividing SEM by the grand mean. The smallest detectable difference (SDD) was calculated to represent the threshold for the smallest detectable change (expressed in absolute and relative units) at the individual level, using the formula SDD = 1.96 × √2 × SEM (Weir, 2005). The significance level was set at p ≤ 0.05 (two-tailed testing).

### 8.2.3 Reliability for length measurements

Based on Table 2, there was no systematic bias in length measurements across all subregions. Furthermore, the CV was considered very low, ranging between 1.8% and 2.2%. Measurement error (SEM) ranged between 0.2 cm and 0.8 cm, corresponding to 1.8% to 2.2% error (SEM%). The intraclass correlation coefficient (ICC) ranged from 0.74 (moderate) to 0.82 (good). Small divergences between tests heavily affect the correlation coefficient and the ICC value due to the clustered lengths around a relatively small distance range. Therefore, the CV and SEM are more relevant metrics for judging length stability.

In conclusion, based on the above, defining the length of the specific subregions can be performed with high assessor reliability. The aim for training a new assessor should be for the CV for lengths to be within 1-3% before a new assessor collects data.

**Table 2**. Test-retest reliability metrics for femur and tibia length (cm) measurements derived from DXA scans

|  | **Test 1** | **Test 2** | **Test 3** | **P** |  | **ICC1.1** | **95% CI** | **CV (%)** | **SEM** | **SEM (%)** | **SDD** | **SDD (%)** |
| --- | --- | --- | --- | --- | --- | --- | --- | --- | --- | --- | --- | --- |
|  |  |  |  |  |  |  |  |  |  |  |  |  |
| Leg straight (total) | 39.3 ± 1.7 | 38.7 ± 1.6 | 38.8 ± 1.9 | 0.075 | Test 1-2 | 0.74 | 0.39-0.90 | 2.2 | 0.8 | 2.2 | 2.3 | 6.0 |
|  |  |  |  |  | Test 2-3 | 0.82 | 0.57-0.96 | 1.9 | 0.7 | 1.8 | 2.0 | 5.1 |
|  |  |  |  |  |  |  |  |  |  |  |  |  |
| Leg straight (50%) | 19.8 ± 0.9 | 19.5 ± 0.8 | 19.5 ± 0.9 | 0.073 | Test 1-2 | 0.74 | 0.39-0.90 | 2.2 | 0.4 | 2.1 | 1.2 | 6.0 |
|  |  |  |  |  | Test 2-3 | 0.82 | 0.57-0.96 | 1.8 | 0.4 | 1.8 | 1.0 | 5.0 |
|  |  |  |  |  |  |  |  |  |  |  |  |  |
| Leg straight (30%) | 12.0 ± 0.5 | 11.8 ± 0.5 | 11.8 ± 0.6 | 0.073 | Test 1-2 | 0.74 | 0.39-0.90 | 2.2 | 0.3 | 2.1 | 0.7 | 5.9 |
|  |  |  |  |  | Test 2-3 | 0.82 | 0.57-0.96 | 1.8 | 0.2 | 1.8 | 0.6 | 5.0 |
|  |  |  |  |  |  |  |  |  |  |  |  |  |
| Table 2 displays the test-retest reliability metrics for the length measurements of tibia obtained from DXA scans across three testing sessions (Test 1, Test 2, and Test 3). Mean lengths with standard deviations, P values, Intraclass Correlation Coefficients (ICC1.1) with 95% Confidence Intervals (CI), Coefficient of Variation (CV), Standard Error of Measurement (SEM), SEM percentage, Smallest Detectable Difference (SDD), and SDD percentage are reported. | | | | | | | | | | | | |

### 8.2.4 Reliability for Lean body mass for the subregion

The intra-assessor placement of subregions demonstrates high reliability and feasibility, with relatively low time consumption for a trained assessor. Becoming a trained assessor can be achieved with just one day of practice.

Based on the reliability analysis, it is recommended that each assessment is performed twice. If a difference of more than 10% is observed between two consecutive assessments, a third assessment should be performed. Furthermore, it is advisable to average the values of Test 1 and Test 2 to provide a more stable estimate of lean body mass. Please refer to Tables 3 and 4 for detailed results.

In the following sections, the reliability data are shown for each subregion.

**Leg Total**

Based on Table 3, there was no systematic bias in the LBM measurements for the total leg across the three assessments. The ICC values were excellent, ranging from 0.99 to 1.00, indicating high reliability during consecutive assessments. The CV% and SEM% for these measurements were very low, ranging from 1.2% to 1.7% and 1.2% to 1.7%, respectively. This measurement error (SEM) corresponded to a range of 24 g to 34 g. These results suggest that the LBM measurements for the total leg were performed with high consistency and reliability. Furthermore, the SDD ranged from 66 g to 94 g, representing a small absolute change in LBM necessary to detect a true difference at an individual level.

**Table 3. Test-retest reliability metrics for lower leg lean body mass (g) measurements derived from DXA scans**

|  | **Test 1** | **Test 2** | **Test 3** | **P value** |  | **ICC1.1** | **95% CI** | **CV (%)** | **SEM** | **SEM (%)** | **SDD** | **SDD (%)** |  |
| --- | --- | --- | --- | --- | --- | --- | --- | --- | --- | --- | --- | --- | --- |
|  |  |  |  |  |  |  |  |  |  |  |  |  |  |
| Leg straight (total) | 2029 ± 459 | 2006 ± 456 | 2010 ± 454 | 0.21 | Test 1-2 | 0.99 | 0.98-1.00 | 1.7 | 34 | 1.7 | 94 | 4.6 |  |
|  |  |  |  |  | Test 2-3 | 1.00 | 0.99-0.1.00 | 1.2 | 24 | 1.2 | 66 | 3.3 |  |
|  |  |  |  |  |  |  |  |  |  |  |  |  |  |
| Leg straight (50%) | 185 ± 38 | 187 ± 35 | 183 ± 40 | 0.57 | Test 1-2 | 0.91 | 0.77-0.97 | 5.8 | 11 | 5.7 | 30 | 15.9 |  |
|  |  |  |  |  | Test 2-3 | 0.93 | 0.82-0.98 | 5.3 | 10 | 5.2 | 27 | 14.5 |  |
|  |  |  |  |  |  |  |  |  |  |  |  |  |  |
| Leg straight (30%) | 90 ± 27 | 96 ± 28 | 92 ± 28 | 0.56 | Test 1-2 | 0.95 | 0.85-0.98 | 6.9 | 6 | 6.8 | 18 | 18.8 |  |
|  |  |  |  |  | Test 2-3 | 0.95 | 0.85-0.98 | 6.9 | 6 | 6.4 | 18 | 18.9 |  |
|  |  |  |  |  |  |  |  |  |  |  |  |  |  |
| Table 3 displays the test-retest reliability metrics for the lean body mass measurements of the femur and tibia obtained from DXA scans across three testing sessions (Test 1, Test 2, and Test 3). Mean lean body mass with standard deviations, P values, Intraclass Correlation Coefficients (ICC1.1) with 95% Confidence Intervals (CI), Coefficient of Variation (CV), Standard Error of Measurement (SEM), SEM percentage, Smallest Detectable Difference (SDD), and SDD percentage are reported. Significant differences (p < 0.05) were not observed between any tests for the lean body mass measurements. | | | | | | | | | | | | | |

**Lower leg 30% and 50%**
For the 30% offset leg, no systematic bias was observed across the three assessments. The ICC values ranged from 0.91 to 0.95 (excellent). The CV% and SEM% ranged from 5.3% to 6.9% and 5.2% to 6.8%, respectively, indicating high reliability. This measurement error (SEM) corresponded to a range of 6 g to 11 g. The SDD ranged from 18 g to 30 g, indicating the minimum change required to detect a true difference in LBM. A misplacement of the sub-region by 1 or 2 pixels can result in a difference of 10-20% between two consecutive measurements. Therefore is it recommened to perform the analysis twice and use an average.
